# Metabolism and upper thermal limits of *Apis mellifera carnica* and *A. m. ligustica*

**DOI:** 10.1007/s13592-014-0284-3

**Published:** 2014-04-02

**Authors:** Helmut Kovac, Helmut Käfer, Anton Stabentheiner, Cecilia Costa

**Affiliations:** 1 Institut für Zoologie, Universität Graz, Universitätsplatz 2, 8010 Graz, Austria; 2 CRA-Consiglio per la Ricerca e la Sperimentazione in Agricoltura, Unità di ricerca di apicoltura e bachicoltura, Via di Saliceto, 80, 42100 Bologna, Italy

**Keywords:** honeybee races, ***carnica***, ***ligustic***a, metabolic rate, lethal temperature, respiration

## Abstract

The Western honeybees *Apis mellifera carnica* and *A. m. ligustica* are closely related subspecies living in neighbouring regions. Metabolism and the upper lethal thermal limits are crucial physiological traits, adapted in the evolutionary process to environment and climate conditions. We investigated whether samples from these two ecotypes differ in these traits. The standard metabolic rate was higher in the *A. m. ligustica* population only at a high temperature (*T*_a_ ~ 40 °C; dVCO_2_ = 12 nl s^−1^; *P* < 0.05), probably due to a higher body temperature (dT_thorax_ = 1.5 °C; *P* < 0.01). The critical thermal maximum of activity and respiration was similar (difference activity CT_max_ = 0.8 °C, respiratory CT_max_ = 1.1 °C). The lethal temperature (LT_50, 8h_) revealed higher tolerance and survival rates of the Ligustica bees (Carnica 50.3 °C; Ligustica 51.7 °C; *P* < 0.02). Results reveal the adaptation of the two subspecies to their historic climate conditions, possibly favouring Ligustica in a warming environment.

## Introduction

The two races of the Western honeybee *Apis mellifera carnica* Pollmann (commonly known as the “Carniolan bee”) and *Apis mellifera ligustica* Spinola (commonly known as the “Italian bee”) are closely related and live in neighbouring climatic regions (Ruttner 1988). The Carniolan bee is the honeybee of the temperate Central European climate region, ranging from the Alps to the Carpaths (Ruttner 1992). The Italian bee is originally native to the Mediterranean South European climate region of the Appenine peninsula, characterized by dry and hot summers. The adaptability of *A. m. ligustica* to a wide range of climatic conditions has favoured its apicultural use throughout the world (Ruttner 1988). Species or races that have a long history of adaptation to a certain environment can be studied to determine their special physiological adaptation (e.g. Vorhees et al. 2013). This condition applies for these two honeybee races.

Temperature is a crucial abiotic parameter in animal life. It influences nearly all physiological and biochemical processes, thus determining a great deal of animal life histories (Huey and Berrigan 2001). Respiration and metabolism are essential basic processes characterizing the ecological potency of a species. Their thermal sensitivity determines relationships between an insect and its environment (Ruel and Ayres 1999). These processes depend strongly on the insects’ actual ambient temperature in their habitat, and they are influenced by mean annual temperature as well as short temperature extremes. The knowledge of the kind of temperature dependence of respiration and its limits is crucial for assessing the species’ ecological potency. Even a small variation in the thermal sensitivity can determine the impacts of variable temperatures on insect performance and energy use (e.g. Williams et al. 2012).

The standard metabolic rate (SMR) or resting metabolism is an important parameter of energy metabolism in insect life. It represents the energetic costs of simple subsistence, determining an individual’s minimum energy requirement under a standardized set of conditions. It is the basis for comparing the relative energy expenditures of particular activities. The SMR allows also the assessment of basic energetic costs across species. Ideally, measurements of SMR are made under constant environmental conditions on individuals of known mass, sex and age that show no external activity (Hack 1997). The SMR of *A. m. carnica* has been thoroughly investigated by Kovac et al. (2007). A dependence of the CO_2_ release on an ambient temperature in a sigmoidal shape has been described. However, for the Italian race, these data are not available. The SMR is an ideal parameter to compare the two honeybee races with regard to their basic energetic expenditure and could reveal special adaptation to the climatic conditions in which they evolved.

The “thermolimit respirometry” is a standardized method for determining the upper and lower critical thermal limits of normal respiratory function (respiratory CT_max_, respiratory CT_min_; Lighton and Turner 2004). A combination of this technique with conventional observation of the critical thermal maximum (activity CT_max_), e.g. using detection of movement by means of a video analysis or infrared diode actography, objectively pinpoints the exact temperature of a short-term physiological failure. It was used by Käfer et al. (2012) for measuring the CT_max_ of the Carniolan honeybee. However, for the Italian subspecies *A. m. ligustica*, these physiological data are missing and therefore were evaluated in this study.

The lethal temperature (LT_50_, the temperature that results in 50 % mortality of an experimental population) has received increased attention in insects in recent years. It does not only provide more ecologically relevant information when studying the survival rate of animals but also provides a reference point to study variation and plasticity of thermal tolerance and traits (e.g. Powell and Bale 2008; Hazell et al. 2010; Hazell and Bale 2011). There are a few papers available concerning the lethal temperature of Western honeybees (e.g. Free and Spencer-Booth 1962; Coelho 1991; Atmowidjojo et al. 1997; Tan et al. 2005). However, most of them did not provide exact information about the investigated race, and the methods and experimental protocols differed strongly. Free and Spencer-Booth (1962) and Coelho (1991) performed experiments with single bees, whereas the others used groups of bees. It is probable that for these reasons, very differing results were obtained. Comparison of the lethal temperature of different races requires simultaneous experiments with the same experimental protocol.

The hypothesis we wanted to test in the present study was whether the two neighbouring races, coming from different climate regions, differ in their thermal traits, especially as far as the upper thermal limits and the metabolism are concerned. In a changing environment due to global climate change, the knowledge of such basal physiological parameters is essential to predict the animals’ capability to cope with predicted future environmental conditions. As no data comparing these crucial thermal traits of the two races are available, adequate experiments were conducted with samples from populations of *A. m. carnica* and *A. m. ligustica*, obtained from their areas of origin (Styria, Austria and Emilia-Romagna, Italy, respectively), both bred by experts for many generations to assure purity of races.

## Materials and methods

### Colonies

Five colonies of *A. m. ligustica* from a honeybee breeder (Eleonora Bergomi) from Emilia Romagna, Italy, and ten *A. m. carnica* colonies of the authors’ (Helmut Kovac) own breeding in Styria, Austria, were donated for the experiments. The experiments were conducted with forager bees, which implies that the individuals were at least 16–20 days old. Bees were caught at the hive entrance after leaving the hive at times where no orientation flight activity occurred (morning and late afternoon).

Morphometric analyses were performed at the CRA-API laboratory. Shortly, 16 characters referring to the front right forewings (11 angles and 5 dimensions) and colour of the second abdominal tergite were used to compare the samples of worker bees from our experimental colonies in the CRA-API reference database (software and statistics are described in Bouga et al., 2011). The analysis confirmed that the samples could be classified as *Apis mellifera carnica* or *A. m. ligustica* as expected.

### Respiration measurement—standard metabolic rate

We measured the bees’ CO_2_ emission using flow-through respirometry as previously described by Kovac et al. (2007). The experiments described here were conducted with the same experimental setup.

Briefly, individual bees were transferred to the laboratory and placed into a respirometry measurement chamber immediately after catching, where they were allowed to move freely. Since experiments lasted overnight, they were provided with 1 M sucrose solution. The brass chamber (outer dimension 6 × 10 × 4 cm; inner dimension 3 × 3 × 2 cm; volume ~18 ml) was immersed in a water bath (Julabo F33) for temperature control (accuracy 0.1 °C). The relative humidity (rH) was maintained at 50 % (see Stabentheiner et al. 2012 for details). The experimental ambient temperature (*T*_a_) for the bees was set to 30, 34, 37 or 40 °C. However, the actual ambient air temperature could deviate slightly from these values because the measurement chamber was not completely submersed to allow observation with an infrared thermography camera (see below). The exact temperature was therefore measured in the respirometry chamber near the bees (within ~1–2 cm) with a thermocouple. Temperature data were recorded at 1-s intervals with an ALMEMO 2890–9 data logger (AHLBORN). Each bee was tested at one temperature and was used for one experiment only.

Carbon dioxide production of the bees was determined with a flow-through respirometry system (Stabentheiner et al. 2012). The insects’ CO_2_ release was measured with a differential infrared carbon dioxide gas analyser (DIRGA; URAS 14, ABB), with an accuracy of ~2 ppm. In order to maximize the system sensitivity (<0.2 ppm), the air was taken from outside the laboratory. Before it entered the reference tube of the DIRGA, the air was pumped through a 10-l container to dampen fluctuations in CO_2_ content, passed the pump and mass flow controllers (0–1000 ml min^−1^, Brooks 5850 S) and then passed another container (5 l) for additional CO_2_ and pressure fluctuation damping. The air was dried by passing it through two Peltier-driven cool traps (10 °C) before it entered the URAS reference and measurement tubes (where it was heated to 60 °C). The airflow in the system was set to 150 ml min^−1^. The volumes (nl) of CO_2_ production reported in this paper refer to standard (STPS) conditions (0 °C, 101.32 kPa = 760 Torr). The CO_2_ production was recorded at 1-s intervals. At the beginning and at the end of each experimental run and at an interval of 3 h during experiments, the gas analyser was calibrated automatically in zero and end point by the use of internal calibration cuvettes, and the data were corrected for any remaining drift or offset (for further methodical details see Stabentheiner et al. 2012).

The lid of the measurement chamber was covered by a transparent plastic film which allowed observing the bees and recording their behaviour and body surface temperature with an infrared thermography camera (ThermaCam SC2000 NTS; FLIR). The plastic film was transparent in the infrared range from 3 to 13 μm. The measured body temperature was calibrated to the nearest 0.7 °C using an infrared emissivity of 0.97 of the honeybee cuticle and a self-constructed Peltier-driven reference source of known temperature and emissivity (for details see Stabentheiner and Schmaranzer 1987; Schmaranzer and Stabentheiner 1988; Stabentheiner et al. 2012). Evaluation of the surface temperatures of head (*T*_hd_), thorax (*T*_th_) and abdomen (*T*_ab_) was done with AGEMA Research software (FLIR Systems Inc.) controlled by a proprietary Excel (Microsoft Corporation) VBA macro. Infrared data were stored digitally on a hard disk at a rate of 5–10 frames s^−1^. Endothermy may increase the energy turnover considerably above the resting level (e.g. Heinrich 1993). Therefore, the thoracic temperature excess (*T*_thorax_ − *T*_abdomen_) was used as a measure to assess the bees’ degree of endothermy. The infrared video sequences allowed quantification of an even small endothermic state of bees over a longer resting period. Bees exhibiting a mean temperature excess of more than 1 °C in the evaluation periods were excluded. In addition, infrared thermography allowed detection of cooling efforts.

The activity and behaviour of the bees was analysed from the infrared video sequences. Our definition of “resting” was no or only small visible signs of activity, i.e. only movements of antennae or single legs are allowed (according to Crailsheim et al. 1999; Stabentheiner and Crailsheim 1999; Stabentheiner et al. 2003; Kovac et al. 2007).

**Figure 1.**
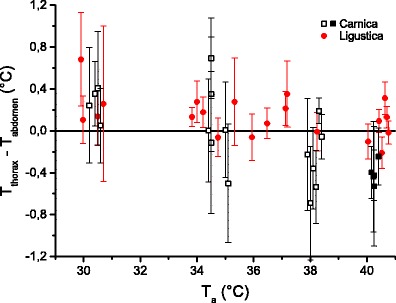
Thoracic temperature excess (T_thorax_ − T_abdomen_) of honeybees, *Apis mellifera carnica* and *A. m. ligustica*, in dependence on ambient temperature (*T*_a_) during resting periods in a respiration measurement chamber. *Open squares* are values from Kovac et al. (2007). *Error bars* represent standard deviation.

**Figure 2.**
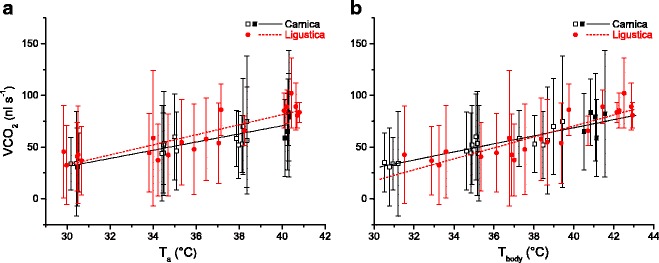
**a** Standard metabolic rate (CO_2_ production) of honeybees, *Apis mellifera carnica* and *A. m. ligustica*, during resting periods in a respiration measurement chamber, **a** in dependence on ambient temperature (*T*_a_), **b** in dependence on body temperature (*T*_body_ = mean of thorax, head and abdomen). *Open squares* are values from Kovac et al. (2007). *Error bars* represent standard deviation. Linear regression functions for **a** Carnica—CO_2_ production = −87.5219 + 3.95603*T_a_, *R* ^2^ = 0.76649, *n* = 21; Ligustica—CO_2_ production = −113.08465 + 4.86236*T_a_, *R* ^2^ = 0.76689, *n* = 21. Linear regression functions for **b** Carnica—CO_2_ production = −87.31312 + 3.90117*T_body_, *R* ^2^ = 0.79567, *n* = 21; Ligustica—CO_2_ production = −141.77306 + 5.30591*T_body_, *R* ^2^ = 0.75657, *n* = 21.

**Figure 3.**
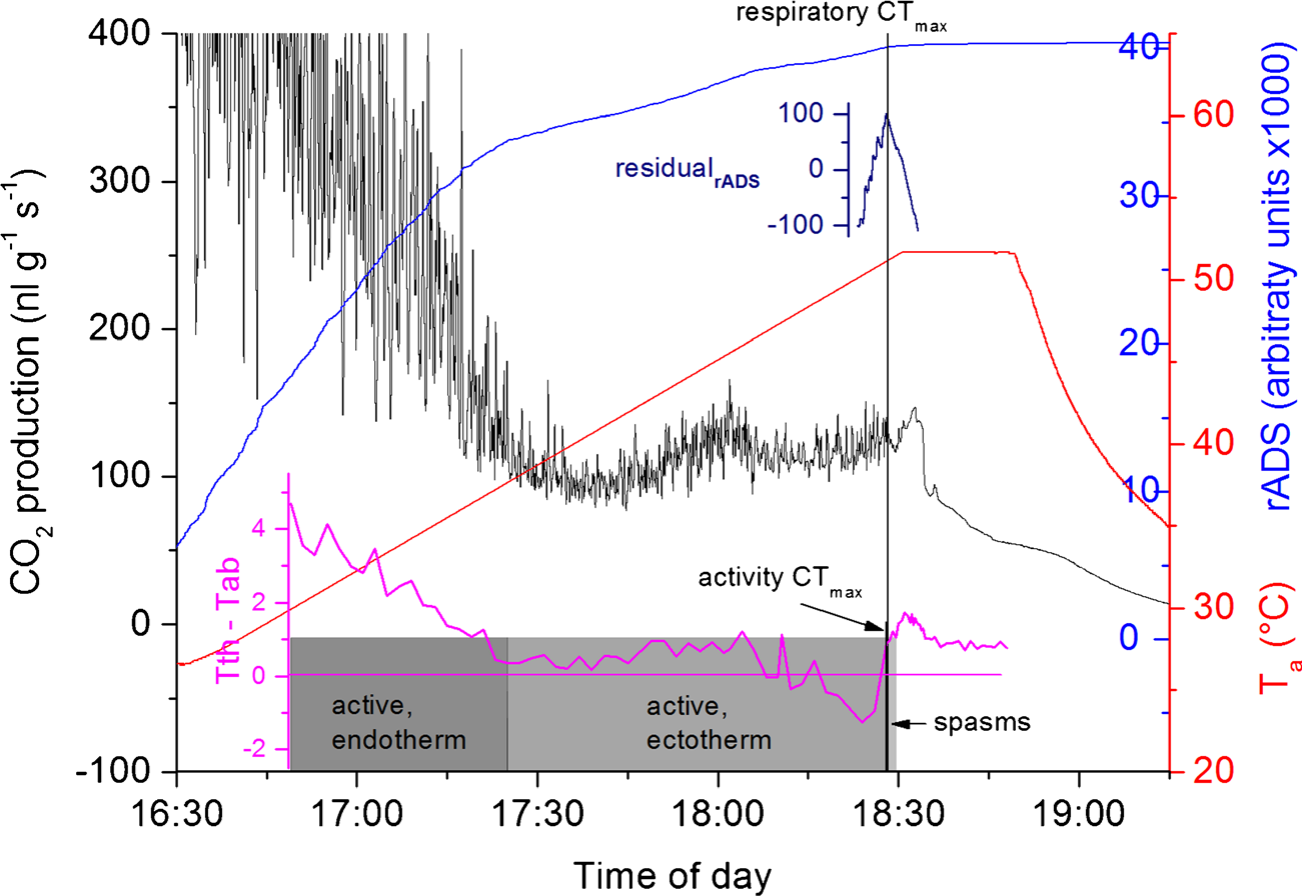
CO_2_ production, activity and thoracic temperature excess of *Apis mellifera ligustica* during a thermolimit experiment. Respiratory CT_max_ = 51.2 °C, activity CT_max_ = 49.1 °C. Activity CT_max_ was indicated by cease of controlled motoric activity (=mortal fall). Residual analysis of ADS indicated the point of respiratory CT_max_ (=cease of cyclic respiration, see “Materials and methods”). The postmortal peak (increase of CO_2_ signal after CT_max_) coincides with a thoracic heating bout starting right after mortal fall.

**Figure 4:**
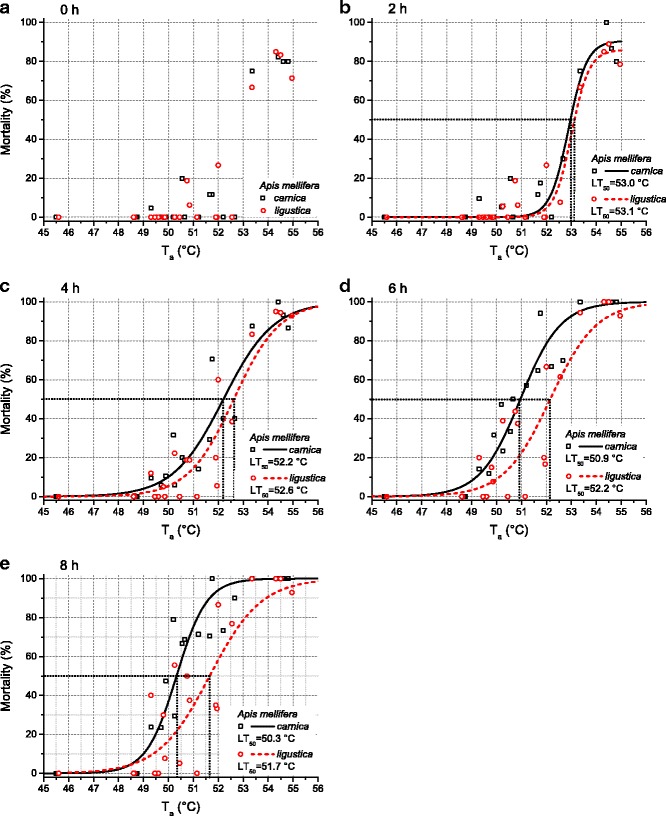
Mortality of honeybees *Apis mellifera carnica* and *A. m. ligustica* in dependence on ambient temperature (*T*_a_) immediately after thermal treatment till 8 h after treatment (**a**–**e**). Twenty-one experiments with 15–25 bees of each race. The lethal temperatures (LT_50_) determined from the sigmoidal curves are indicated. Curves were best-fitted with a sigmoidal function (mortality = a/(1 + b*exp^-kTa^). Parameters for functions; **a** Function could not be fitted. **b** Carnica, *a* = 90.53527, *b* = 2.07583E62, *k* = 2.71334; Ligustica, *a* = 85.98757, *b* = 1.95105E65, *k* = 2.83602. **c** Carnica, *a* = 100, *b* = 3.00187E22, *k* = 0.99141; Ligustica, *a* = 100, *b* = 8.09868E24, *k* = 1.0899. **d** Carnica, *a* = 100, *b* = 3.07402E27, *k* = 1.24218; Ligustica, *a* = 100, *b* = 2.36776E24, *k* = 1.07619. **e** Carnica, *a* = 100, *b* = 1.26685E36, *k* = 1.65179; Ligustica, *a* = 100, *b* = 5.51212E21, *k* = 0.96916.

For further data analysis, the evaluated resting phases were divided into 10-min intervals. In some individuals at a high *T*_a_, the duration of resting phases decreased to such an extent that we had to reduce the interval to a minimum of 5 min. For the evaluated intervals, the mean CO_2_ production rate (VCO_2_) was calculated. A respiration cycle was defined from the beginning of one burst of CO_2_ release until the next one. The CO_2_ release volume for each respiration cycle was calculated by the integration of single bursts. Data analysis and statistics were done in Excel (Microsoft Corporation) with custom-made peak-finding formulas and VBA macros and with Origin software (OriginLab Corporation) and Stathgraphics Centurion XVI (StatPoint Technology Inc.). In Figures 1, 2, 3, and 4, mean values are given with their standard deviations (SD).

The SMR data of *A. m. carnica* bees were taken from the paper of Kovac et al. (2007), with the exception of the measurements at 40 °C, which were made during the same experimental series as *A. m. ligustica* bees in 2012 and 2013.

### Critical thermal maximum (CT_max_)

Single bees were caught at the hive entrance and immediately placed into the respirometry measurement chamber. The combination of respirometry data and activity detection has shown the most accurate results in previous studies concerning the upper thermal maximum (Klok et al. 2004; Lighton and Turner 2004; Stevens et al. 2010; Käfer et al. 2012). Thus, respiration and activity as well as body surface temperatures were assessed simultaneously via flow-through respirometry and infrared thermography as described in Section 2.2. The bees’ critical thermal maximum (CT_max_) was evaluated following a standardized method of driving a temperature increase from 25 to 55 °C at a dT = 0.25 °C min^−1^ (see e.g. Chown et al. 2009; Stevens et al. 2010; Terblanche et al. 2011). The CT_max_ was defined via the observation of activity as cease of controlled motoric activity (“mortal fall” or activity CT_max_; e.g. start of muscle spasms, compare Hazell et al. 2008; Klok and Chown 1997; Lighton and Turner 2004; Lutterschmidt and Hutchison 1997) and via thermolimit respirometry (respiratory CT_max_) as cease of cyclic gas exchange (CO_2_ release; Lighton and Turner 2004). The absolute difference sum of CO_2_ production (respiratory ADS, rADS) is a measure of cumulative dynamic variability (Lighton and Turner 2004). To determine the respiratory CT_max_ more accurately, the inflection point of the rADS residual values from 10 min before to 10 min after the suggested activity CT_max_ was determined. This inflection point helps to determine the minute point of the respiratory CT_max_. For detailed information on the experimental procedure, see for example Stevens et al. (2010), Käfer et al. (2012), Stabentheiner et al. (2012) and chapter 6 in Hartfelder et al. (2013).

### Lethal temperature

Bees were caught in the morning at the hive entrance. For each trial, groups of bees (15–25 individuals) of the two races were separated in a divided wire mesh cage (10 × 10 × 5 cm) and put into an incubator. During the experiments, the bees were provided with 1 M sucrose solution. After 5 min at 30 °C to ensure uniform starting conditions, the temperature was increased at a rate of about 0.4 °C per minute until the experimental target temperature (46–56 °C, 21 different trials) was reached. The bees remained for 5 min at the maximum target temperature to allow all individuals to equilibrate (compare Powell and Bale 2008). After that, the temperature in the incubator was rapidly reduced to the initial temperature of 30 °C. The bees remained for further 8 h in the incubator at this temperature. Survival was defined by bees moving either spontaneously or in response to a gentle contact stimulus, assessed at an interval of 2 h. The air in the incubator was moistened with a wet cloth (mean relative humidity during thermal treatment was ~30 %). The temperature was measured with thermocouples in the centre of each section of the cage and additionally ~2 cm outside the cage with a NTC-sensor. The measured temperature inside and outside of the cage differed noticeably. As the bees sat mostly at the wire mesh of the cage during the experiments, we used the mean value between the inside and outside for calculating the actual experimental temperature. Data were stored every second with an ALMEMO 2890–9 data logger (AHLBORN). The percentage of the bees’ mortality rate was plotted vs. the tested experimental temperature; and from the calculated sigmoidal curve, the lethal temperature (=LT_50_, the temperature that results in 50 % mortality) was determined. The significance was tested for the linear range of values (without a lowest value) with an ANOVA.

## Results

### Standard metabolic rate

#### Activity and endothermic state

Infrared video sequences enabled us to evaluate the behaviour and the body temperature of the bees. Although the bees stayed overnight in the dark measurement chamber, they were not always calm. They were sometimes moving, walking or feeding. Some individuals were not inactive for 10 consecutive minutes, especially at high ambient temperatures (~40 °C). Therefore, we had to reduce the evaluation intervals of the resting phases to 5 min in these cases.

The body temperatures measured in *A. m. ligustica* bees (from here on referred to as “Ligustica”) were compared with those measured in *A. m. carnica* bees (from here on referred to as “Carnica”) by Kovac et al. (2007) or with new measurements at *T*_a_ ~ 40 °C. Mostly, the bees were ectothermic or showed only a little endothermic activity and had a little thoracic temperature excess (*T*_thorax_ − *T*_abdomen_ < 0.4 °C; Carnica *n* = 21, Ligustica *n* = 21; Figure 1). The Ligustica bees had, on average, a thorax 0.2 ± 0.47 °C warmer than their abdomen, whereas the Carnicas’ thorax was 0.16 ± 0.55 °C cooler than their abdomen (Figure 1). ANOVA suggested that the excess temperature of the two races depended on ambient temperature (*P* < 0.01; *F*-quotient = 12.03) and differed from each other (*P* < 0.01; *F*-quotient = 7.9). However, a more detailed view of the results revealed that the values below an ambient temperature of 31 °C and between 33.5 to 38.5 °C did not differ from each other (*P* > 0.05; *t* test), but the values above 40 °C were statistically different (Carnica—*T*_thorax_ − *T*_abdomen_ = −0.41 ± 0.41 °C; *n* = 5; Ligustica—*T*_thorax_ − *T*_abdomen_ = 0.02 ± 0.21 °C; *n* = 6; *P* < 0.01, *t* test). The absolute thorax temperature at *T*_a_ ~ 40 °C differed also between Carnica and Ligustica (Carnica—*T*_thorax_ = 41.1 ± 0.71 °C; *n* = 5; Ligustica—*T*_thorax_ = 42.6 ± 0.57 °C; *n* = 6; *P* < 0.01, *t* test)

#### Metabolic rate

The metabolic measurements in Ligustica were compared with those in Carnica from Kovac et al. (2007) or with new measurements at *T*_a_ ~ 40 °C. The resting or standard metabolic rate (SMR) showed a strong dependence on ambient and body temperature (*P* < 0.0001; *n* = 21; *F*-quotient = 131.44 and 125.54, respectively, ANOVA; Figure 2a, b). It differed between the two races only in dependence on ambient temperature (*P* < 0.05; *n* = 21; *F*-quotient = 6.27 and 0.41, respectively, ANOVA). The variation between bees and within individual bees was rather high. The mean SMR (derived from the regression lines) increased from 32.6 nl s^−1^ (*T*_a_ = 30 °C) to 70.6 nl s^−1^ (*T*_a_ = 40 °C) in the Carnica and from 32.9 nl s^−1^ (*T*_a_ = 30 °C) to 81.3 nl s^−1^ (*T*_a_ = 40 °C) in the Ligustica bees. For a further analysis, the values were divided in different ranges (*T*_a_ < 33 °C, 33–40 °C, >40 °C). Only at high ambient temperatures of >40 °C the SMR differed significantly between the two races (Carnica—VCO_2_ = 75.7 ± 42.38 nl s^−1^; *n* = 5, Ligustica—VCO_2_ = 87.6 ± 20.63 nl s^−1^; *n* = 6; *P* < 0.05, *t* test). The correlation of the SMR with the body temperature (mean of head, thorax and abdomen) revealed a nearly identical result for both races (*P* = 0.5248; Figure 2b).

### Critical thermal maximum (CT_max_)

The bees were active and agitated when they were inserted into the measurement chamber. They calmed down after reaching an ambient temperature of about 35 °C and were again more active when the temperature exceeded 40 °C. At high temperatures, they often exhibited cooling behaviour with regurgitated fluid droplets. Coordinated body movement ceased with the mortal fall. Figure 3 shows a representative thermolimit experiment for determining the critical thermal maximum (CT_max_) of activity and respiration of a Ligustica bee. The averaged values of mortal fall provided the knockdown temperature (Klok et al. 2004; Stevens et al. 2010) or activity CT_max_ of 49.2 ± 2.0 °C for the Carnica bees and 50.0 ± 1.0 °C for the Ligustica bees (statistical details in Table 1). After the initial high activity and therefore metabolic rate, the CO_2_ trace showed a typical progression, followed by a distinct postmortal peak (plateau) after the respiratory CT_max_. Averaged values of the respiratory CT_max_ amounted to 48.8 ± 2.2 °C for the Carnica bees and 49.9 ± 1.1 °C for the Ligustica bees. Although the mean activity and respiratory CT_max_ in *A. m. ligustica* were somewhat higher than in *A. m. carnica*, differences were not statistically significant (difference activity CT_max_ = 0.8 and respiratory CT_max_ = 1.1 °C, *P* > 0.05, *t* test; see Table 1).

**Table I:** Critical thermal maximum (CT_max_) of activity and respiration of honeybees, *Apis mellifera carnica* (Car) and *A. m. ligustica* (Lig). Means were compared with *t* test. *SD* standard deviation, *N* number of experiments (bees)

	Activity CT_max_						Respiratory CT_max_				
	mean	SD	max	min	*N*	*P*	mean	SD	max	min	*N*
*A. m. carnica*	49.2	1.96	51.8	43.8	20	0.5689	48.8	2.21	51.9	43.9	20
*A. m. ligustica*	50.0	1.01	51.7	48.2	20	0.8572	49.9	1.09	51.9	48.1	20
*P* (Car vs. Lig)	0.1176						0.0530				

The mean maximum thoracic temperature we measured during the time the postmortal plateau appeared was nearly the same in Carnica and Ligustica (Carnica *T*_th_ = 52.3 ± 0.6 °C, Ligustica *T*_th_ = 52.4 ± 1.0 °C). The absolute maximum was measured in a Ligustica bee with 54.4 °C. The thoracic temperature excess over the abdomen also showed a peak at the same time as the postmortal respiratory peak appeared. The amount of the thoracic heating bout was quite similar for the two subspecies and not statistically different (dT_thorax − abdomen_ maximum–dT_thorax − abdomen_ minimum before peak, Carnica—2.2 ± 0.7 °C, *n* = 18; Ligustica—1.8 ± 1.2 °C, *n* = 20; *P* > 0.05, *t* test). No differences were detected in the duration of the cooling behaviour during the experimental runs (cool tongue visible in the thermograms). Durations were 21 min 44 s (SD = 6 min 31 s) in Carnica and 27 min 04 s (SD = 9 min 40 s) in Ligustica (*P* > 0.05, *t* test).

### Lethal temperature

The mortality of the temperature-treated bees displayed a time-dependent course. It was low immediately after the treatment and increased with progressing observation time (Figure 4). From zero until 4 h after the end of treatment, no difference was detectable between the two races’ mortality. However, after 6 and 8 h, significant differences appeared. A comparison of the linear course of the relative mortality values (without a lowest value of ~45 °C) revealed a significant difference between the two races (6 h—*P* = 0.0112, *F*-quotient = 7.12; 8 h—*P* = 0.0190, *F*-quotient = 6.02; ANOVA). The lethal temperature (=LT_50_, the temperature that results in 50 % mortality) was determined from the mortality curves (Figure 4), derived by fitting a sigmoidal function [(mortality = a/(1 + b*exp^-kTa^)]. Eight hours after, the temperature treatment parameters were *a* = 100 and *b* = 1.26685E36 and *k* = 1.65179 in Carnica and *a* = 100 and *b* = 5.51212E21 and *k* = 0.96916 in Ligustica. The lethal temperature after 8 h, derived from the sigmoidal curves, was for the Ligustica 1.4 °C higher than for the Carnica bees (Carnica—LT_50, 8h_ = 50.3 °C; Ligustica—LT_50, 8h_ = 51.7 °C; *N* = 21 trials).

## Discussion

In the evolutionary process, insects have evolved behavioural and physiological adaptations to cope with short-term exposure to extreme temperatures. Metabolism and the upper lethal limits are crucial physiological traits. They are, besides other biotic and abiotic parameters, responsible for the distribution of insects. We investigated these traits in two populations of neighbouring and closely related honeybee subspecies (*Apis mellifera carnica* and *A. m. ligustica*) living in different climate regions. Honeybee colonies for the experiments were chosen from commercial queen breeders, situated in the centre of the original distribution area of each subspecies and who have been selecting colonies for a long time, without introducing genetic material from different sources. Indeed in Southern Austria, in the whole area of Styria and Carinthia by law beekeepers are allowed to keep only *A. m. carnica* to ensure its protection. We are aware of the fact that the colonies (of each subspecies) originated from one population each, and therefore results have validity, in the strict sense of the word, only for these populations. Nevertheless, our experiments suggest that there may be differences among different subspecies due to adaptation to different climates. Our experiments provide a profound investigation on these physiological traits in honeybees. These measurements with several standard methods established in insect research can lead to a standard in future honeybee research.

The standard metabolic rate (SMR), as a measure of the basal energy turnover, was investigated at high ambient temperatures (*T*_a_ ~ 30-40 °C, Figure 2). The new data of Carnica (*T*_a_ ~ 40 °C) were quite well in the trend of the measurements of Kovac et al. (2007) included in this comparison (Figure 2). For a great part of the tested temperature range, the two races did not differ in their SMR. However, at the highest tested *T*_a_ (~40 °C), the Ligustica bees had a higher CO_2_-production rate than the Carnica bees (dVCO_2_ = 11.9 nl s^−1^, *P* < 0.05; Figure 2). The Ligustica bees’ thorax temperature and thoracic temperature excess were also higher (differences between races—dT_th_ = 1.54 °C, *P* < 0.01; dT_th − ab_ = 0.42 °C, *P* < 0.01; Figure 1). We thus assume the higher metabolic rate to have been caused by the higher body temperature of the Ligustica bees. Our measurements of body temperature suggest that the difference in body temperature (and thus metabolic rate) was not a result of endothermic activity of the Ligustica bees, but of more cooling efforts by means of regurgitated liquid droplets of the Carnica bees. The negative thoracic temperature excess in the Carnica bees at this high ambient temperature (Figure 1) suggests this. Evaporative cooling with water droplets is a common behaviour of honeybees at high ambient temperatures to reduce body temperature (Heinrich 1980, Kovac et al. 2007). Based on our results, we assume that the Ligustica bees tolerate higher body temperatures than the Carnica.

As expected from earlier measurements in Carnica honeybees (Kovac et al. 2007), the resting metabolism of Ligustica bees showed a strong dependence on ambient and body temperatures (Figure 2). In active, flying honeybees, Harrison and Hall (1993) showed that the mass-specific metabolic capacities in African bees (*A. m. scutellata*) were higher than in European bees. The greater thorax-specific capacity in African bees suggests a higher oxidative capacity in their flight muscles compared to European bees. The authors presume that higher metabolic and flight capacities may contribute to African bees’ competitive advantages in the tropics and that differences in flight capacity or metabolism may be an important component of fitness differences among honeybee subspecies. However, in the resting metabolism of our investigated subspecies, we could not detect such differences, both in the normal colony thermal range (30–36 °C; Bujok et al. 2002, Kleinhenz et al. 2003, Stabentheiner et al. 2010) and at temperatures of thermal challenge (36–40 °C).

Our thermolimit respirometry experiments provided results concerning the short-term upper critical thermal maximum of activity and respiration (Table 1). These two thermal limits did not differ from each other, neither in the Carnica (dT = 0.4 °C, *P* > 0.05) nor in the Ligustica bees (dT = 0.1 °C, *P* > 0.05), thus, suggesting that in future experiments, measurement of one of the two is sufficient. These parameters showed a tendency towards a higher respiratory CT_max_ in the Ligustica bees in comparison to the Carnica population sample though the difference with *P* = 0.053 cannot be considered statistically significant. The activity CT_max_ did not differ significantly (dT = 0.8 °C, *P* = 0.12). This finding is confirmed by the experiments concerning the lethal temperature (LT_50_), where there was no immediate difference in survival after the temperature treatment. In the following hours, however, the mortality increased stronger in the Carnica bees, as became manifest after 6 h. These experiments revealed a 1.4 °C higher LT_50_ for the Ligustica bees 8 h after the end of treatment (Carnica—LT_50_ = 50.3 °C; Ligustica—LT_50_ = 51.7 °C; *P* < 0.02; Figure 4), showing that the damage due to thermal treatment was delayed. There was no immediate, but a medium-term, harmful effect on the bees’ survival rate. This points out that only survival analysis can completely uncover total thermal damage to the honeybee society. The dynamic determination of the CT_max_, by contrast, uncovers the short-term upper thermal limits. A main benefit of this standard method allows a rather fast comparison with other populations, honeybee races or insects. Since our investigation showed that activity ceases at quite the same temperature as controlled respiration (see also Klok et al. 2004; Lighton and Turner 2004; Stevens et al. 2010; Käfer et al. 2012; and others), determination of the activity CT_max_, which is easier to accomplish in standard laboratories, seems sufficient.

We do not know whether the Ligustica bees’ higher LT_50_ is the result of physiological traits or behavioural differences, or both. As the bees were provided with sufficient liquid food during experiments, they were able to perform cooling measures. Further experiments with detailed video and body temperature observations will be needed to decide whether cooling activity is less frequent in Ligustica. It has also to be clarified whether there are differences in physiological adaptation, e.g. expression of heat shock proteins.

Free and Spencer-Booth (1962) showed that the survival of bees at high temperatures depends on the duration of exposure and the relative humidity of the air. At the higher temperatures, they survive for short periods only at low relative humidity because they can cool themselves by evaporative cooling via regurgitation of water or nectar droplets (Heinrich 1980, Cooper et al. 1985). These conditions were given in our experiments concerning lethal temperature, as the bees were provided with sucrose solution and the relative humidity was low (~30 % rH). Abou-Shaara et al. (2012) investigated the tolerance of Yemeni honeybees (adapted to harsh conditions) and Carniolan honeybees to various temperature and relative humidity gradients in Saudi Arabia. Bees unable to right themselves immediately were classed as intolerant. Results showed that temperature had a higher effect than relative humidity on worker survival. The authors commented that the response of the two races in the studied treatments was somewhat similar and that only under extreme conditions of elevated temperature or low humidity, Yemeni honeybees showed a higher tolerance than Carniolan honeybees. In contrast to our thermolimit experiments, they found very high temperature intolerance values, which only could be explained by different methods (Carniolan honeybees 57.5 °C, Yemeni honeybees 61.0 °C). When temperature is increased to a limit, the starting temperature and rate of increase can significantly influence results, as illustrated by estimates of knockdown temperature in the tsetse fly, *Glossina pallipides*, with the slowest rate of thermal change producing the lowest estimate (e.g. Terblanche et al. 2007; 2011; see also Chown and Nicolson, 2004). This could be the reason for the very high values obtained by Abou-Shaara et al. (2012), because they started at 30 °C and increased to 60 °C at a rate of 0.5 °C per minute, whereas we increased the temperature from 25 °C to 55 °C at a rate of 0.25 °C per minute.

Atmowidjojo et al. (1997) compared the temperature tolerance of feral and domestic honeybees (*Apis mellifera*) in the Arizona desert in different seasons and at different ambient temperatures and found the feral honeybees to be more tolerant to high temperatures than domestic bees. The highest critical thermal maximum (temperature at which animals fail to undergo an immediate righting response) of the feral bees was 50.7 °C, which resembles the mean value of the Carnica bees (50.4 °C). However, the values of these bees, adapted to the Arizona desert, seem to be implausibly low, as most of the measured thermal maxima were below 45 °C. There are several investigations of honeybee body temperature showing that the thoracic temperatures may exceed 45 °C due to their endothermic heat production (e.g. Heinrich 1979; Cooper et al. 1985; Stabentheiner et al. 2002, 2007; Kovac et al. 2010). Coelho (1991) investigated the upper thermal tolerance limit of drones and workers and found the lethal thorax temperature to be statistically indistinguishable between workers (52.2 °C) and drones (51.0 °C). The lethal temperature of workers (*Apis mellifera* L.) was 0.5 °C higher than that of our Ligustica bees. However, this could also be due to the different methods. Tan et al. (2005) reported differences in the lethal temperature of the Eastern honeybee *Apis cerana* and the Western honeybee *Apis mellifera* (*A. cerana* 50.7 °C and *A. mellifera* 51.8 °C, respectively). Their *Apis mellifera* had nearly the same lethal temperature as our Ligustica bees. Unfortunately, no information about the bee race is given in this study.

In conclusion, our experiments and the evaluation of available literature call for a standardisation of methods and experimental protocols. In this study, we measured several important physiological parameters with standard methods and thus provide a basis for future studies. Furthermore, we investigated for the first time the difference in heat tolerance in populations of two European races of the Western honeybee. We found differences in several traits, suggesting that *A. m. ligustica* populations can tolerate a high temperature exposure better than *A. m. carnica* populations. This surely represents a feature of adaptation of *A. m. ligustica* populations to the warmer environment of the South European climate. However, heat tolerance is probably just one aspect of the complex process of adaptation, which can contribute to higher survival and performance in the areas of origin of populations, as has been highlighted in recent studies across Europe (Costa et al. 2013, Büchler et al. 2014; Hatjina et al. 2014 in press). Other features like the ability of synchronization and tuning of the breeding behaviour with food availability could be also a limiting factor. The distribution of Ligustica bees all over the world in the wake of human introduction demonstrates its good ability to adapt to different climatic conditions (Ruttner 1988). This could also be a benefit in the chance of survival in a changing environment due to climate warming. Increasing mean annual temperature could enable them to expand their original European distribution range into the north. Additionally, their ability to cope better with high temperature extremes may be an important component of fitness benefit in the future struggle for survival in Central Europe in a changing environment with more pronounced weather extremes due to climate change (IPCC 2013).
